# On Local Instability of Deep-Profiled Facings in Sandwich Panels

**DOI:** 10.3390/ma18225162

**Published:** 2025-11-13

**Authors:** Zbigniew Pozorski, Jolanta Pozorska, Zuzana Murčinková, Dawid Cekus

**Affiliations:** 1 Institute of Structural Analysis, Faculty of Civil and Transport Engineering, Poznan University of Technology, Piotrowo 5, 60-965 Poznań, Poland; 2 Institute of Mathematics, Faculty of Control, Robotics and Electrical Engineering, Poznan University of Technology, Piotrowo 3a, 60-965 Poznań, Poland; jolanta.pozorska@put.poznan.pl; 3 Faculty of Manufacturing Technologies with Seat in Prešov, Technical University of Košice, Bayerova 1, 080 01 Prešov, Slovakia; zuzana.murcinkova@tuke.sk; 4 Department of Mechanics and Machine Design Fundamentals, Faculty of Mechanical Engineering, Czestochowa University of Technology, Dąbrowskiego 73, 42-201 Częstochowa, Poland; dawid.cekus@pcz.pl

**Keywords:** sandwich panels, local instability, wrinkling, strain energy

## Abstract

**Highlights:**

**What are the main findings?**

- The actual increase in load-bearing capacity was obtained for a facing bandwidth of less than 10 cm.

**What are the implications of the main finding?**

- The critical stress in compression of a thin facing band resting on a flexible core was evaluated.
- The benefits of changing the geometry and support stiffness of the facing bands were determined.

**Abstract:**

This study focuses on the problem of local instability of sandwich panels, which consist of two thin but relatively stiff facings and a thick but shear-deformable core. Such structures are commonly used in civil engineering and in the aerospace, aviation, and automotive industries. A case is presented in which one of the facings is deep-profiled. Due to typical mechanical or thermal interactions, this facing is subjected to compression. The thick core of the sandwich panel plays a stabilizing role. However, at a specific critical load, local stability is lost, representing a typical form of damage that occurs in sandwich panels. In the case of a deep-profiled facing, the geometry of the facing must also be taken into account, specifically the fact that the bends resulting from profiling constitute a significant limitation to its deformation. In this study, expressions are derived that enable the determination of the critical (wrinkling) stress, taking into account the geometry of the compressed facing bands and various boundary conditions defined along their edges. The energy approach is used to solve the problem. The presented solution to the problem of local instability is illustrated using examples. The obtained results indicate that the use of narrow bands is particularly effective while also allowing for determination of the maximum benefits resulting from deep profiling of the facings. This information is essential when considering changes to the geometry of industrially produced sandwich panels or when optimizing the load-bearing capacity of individual sandwich elements.

## 1. Introduction

This study focuses on sandwich panels, which consist of two thin, stiff facings and a thick but shear-deformable core. The facings, usually made of metal, serve as protective layers. From a mechanical point of view, their main task is to transfer the normal stresses resulting from the bending of the panel. The core of the panel provides thermal insulation and is made from materials with lower elastic moduli, such as polyisocyanurate foam, expanded polystyrene, or mineral wool. The core is susceptible to deformation, but transfers shear stresses and separates the facings, giving the panel flexural rigidity.

**Figure 1:**
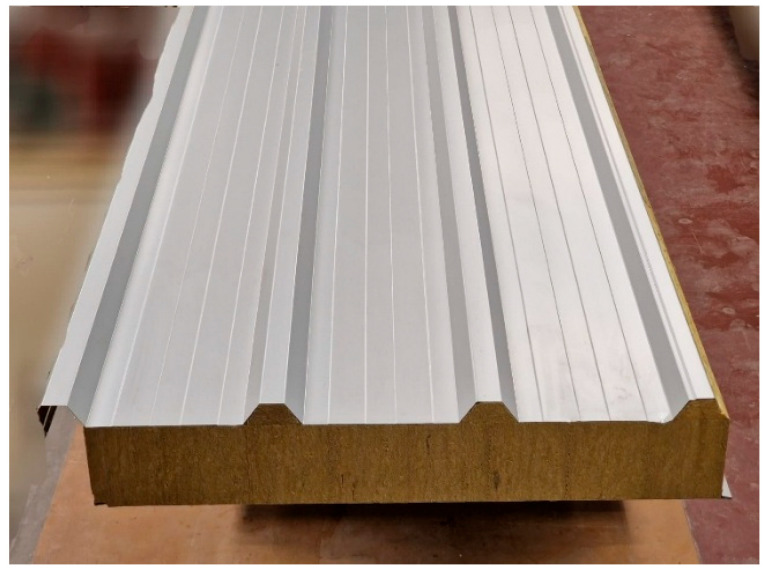
Sandwich panel with deep-profiled steel facing.

Sandwich panels are a universal solution that combines thermal insulation, lightness, durability, and quick installation. These types of elements are widely used in construction and many other industries, including aviation, space, and automotive. An example of a sandwich panel used for roofing is shown in Figure 1.

The sandwich elements can fail in various ways, but their load-bearing capacity is most commonly limited by local instability, often in the form of wrinkling of the compressed facing. The compressive stress may result from axial compression, bending moments, or thermal effects. Accurately determining the panel’s load-bearing capacity requires identification of the critical stress level at which wrinkling occurs.

For decades, researchers have been studying how to predict wrinkling stress in sandwich structures. This is a crucial issue because it plays a critical role in ensuring structural stability. One of the earliest analytical solutions was proposed in [1], where the energy method and a linear decay function were used to describe the core deformation. This approach was developed in [2] by introducing an exponential decay function along the core thickness. The local buckling problem was solved here for both 2D and 3D problems, but the facing deformation function in each direction was sinusoidal. In [3], a different approach was used, involving modeling the wrinkling problem using differential equations. The 2D problem was solved by applying the Airy stress function.

The classical approaches presented above are still used and have been extended to more complex problems. The issue of wrinkling of sandwich panels with composite facings under biaxial load was investigated in [4]. The local instability of a thin orthotropic layer was analyzed in [5]. The effects of anisotropy and multiaxial loading on the wrinkling of sandwich panels were discussed in [6]. Another interesting problem is local instability in the case of a bi-modular core [7]. The stiffness of such a core differs in tension and compression, which obviously affects the structure’s behavior. The effect of transverse core compressibility was considered in [8], where the static and dynamic responses of layered structures, including the wrinkling phenomenon, were examined. A similar problem concerning local dynamic instability, i.e., large-amplitude, small-wavelength lateral vibrations, was addressed in [9], where the criterion for the onset of dynamic wrinkling and a critical value of the facing damping coefficient were presented for the elastic foundation model. The damping of sandwich composites, expressed as the logarithmic decrement, damping ratio, and loss factor, can be determined experimentally using the free vibration decay effect, evaluated using the logarithmic decrement and half-power bandwidth methods [10].

The application of the extended high-order sandwich panel theory to the problem of sandwich panels wrinkling was presented in [11]. The authors demonstrated good agreement between the theoretical and experimental results. The behavior of structures with a functionally graded core material in the context of local instability was analyzed in [12]. A new method for predicting the wrinkling stress in sandwich panels was proposed in [13]. This method can be easily applied to compute the wrinkling stress of panels with functionally graded material cores.

Scientific research on the wrinkling of thin facings is still of great interest because the problem of local instability is important in many applications. The issue of local buckling in the context of using sandwich panels in light aviation was discussed in [14], where among other things, experimental methods and failure scenarios were examined. The analysis of sandwich structures, which have been widely applied in the wings and horizontal tails of aircraft, was presented in [15]. The study proposed a reasonable strategy for resisting wrinkling deformation of sandwich structures. New applications of sandwich structures often necessitate the development of new materials or structural solutions. In [16], structures made of unidirectional carbon/epoxy facings and aluminum honeycomb and closed-cell polyvinylchloride foam cores were studied. The experimental results confirmed that wrinkling failure is prevalent in cases of low through-thickness stiffness and long beam spans. According to [17,18], the influence of production parameters and operating conditions necessitates extensive experimental studies to accurately predict sandwich panel damage, enabling the reliable application of advanced models and failure criteria.

Slightly different structures, namely those made of printed polylactic acid, were studied in [19]. Three honeycomb core topologies were subjected to bending: conventional, re-entrant auxetic, and chiral. Different failure mechanisms were identified for each of these structures, including local loss of stability. The problem of the compression behavior of sandwich elements was also discussed in [20]. In this case, an aluminum honeycomb structure filled with ethylene vinyl acetate copolymer foam was investigated.

Both new applications of sandwich panels and the introduction of new material solutions require detailed strength analyses. This is particularly applicable to products manufactured on a large scale. This article addresses the problem of damage to the facings of deep-profiled panels. The inspiration for conducting this research was the expectations of sandwich panel manufacturers regarding the effectiveness of introducing deep-profiled facings. The solution presented in this paper considers the impact of all the most significant parameters of sandwich panels on the wrinkling of the metal facing when it rests on a 3D core. A novelty in relation to previous theoretical solutions is the use of a two-dimensional model of the facing (band) with the simultaneous possibility of introducing various boundary conditions (including elastic support) on the longitudinal edges of this band. Previous classical solutions [1,2,3] were based exclusively on the sine deformation function. The introduction of different support conditions necessitates the use of different functions, and the corresponding critical loads (and stresses) differ significantly from those presented in previous solutions, as discussed in this paper.

## 2. Formulation of the Problem

The sandwich panel shown in Figure 1 is typically used as a roof covering element placed on purlins. In such cases, the panel is subjected to loads including its own weight, wind and snow actions, and, importantly, various thermal impacts. As a result of these loads, a complex state of stress develops in each component of the sandwich panel (top face, core, and bottom face). In the case of tension or compression, it dominates along the direction of the panel, and this results from the bending of the sandwich panel. The purpose of the core is to transfer shear stresses and ensure cooperation between the facings.

**Figure 2:**
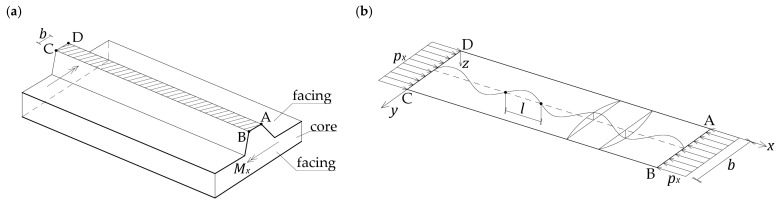
A band of facing (ABCD) being analyzed: (**a**) a section of a deep-profiled sandwich panel, and (**b**) deformations of a facing band subjected to uniform, unidirectional compression.

This article focuses on the deep-profiled facing (top facing in Figure 1). When the facing is stretched, its load-bearing capacity is relatively high. When the facing is compressed, the problem of local instability occurs. This is due to the high slenderness of the element. The typical thickness of the facing is 0.5 mm, whereas the span of the panel is approximately 3 m. It is also noteworthy that bending the panel shown in Figure 1 produces the highest stresses in regions furthest from the center of the neutral plane. Specifically, in the case of a deep-profiled facing, this stress occurs in the narrow ridge bands. One such band is marked in Figure 2a (band ABCD).

In this paper, the case of a perfectly flat band ABCD, which is uniformly unidirectionally compressed in its plane, is considered. It is assumed that this band rests on an infinite core. Although this assumption may seem controversial at first, numerous studies have confirmed that core deformations disappear very quickly across their thickness. From a practical point of view, it can be assumed that deformations at a depth greater than 2 cm measured from the surface of the compressed facing are negligible [21,22].

As a result of uniaxial compression of the ABCD band of width b, at a specific load $p_{x}$, local instability occurs, exhibiting the form of periodic wrinkling in the *x*-direction (Figure 2b). The edges of the band limit the form of deformation in the *y*-direction. The critical load $p_{x}$ is sought, which causes wrinkling of the facing. Determining this load is essential because the wrinkling phenomenon is the primary failure mechanism of a sandwich panel. The key issue of this study is assessment of the influence of the bandwidth b and support conditions on the longitudinal edges of the analyzed band (BC, AD) on the critical load.

In the analysis, it was assumed that the materials of the facings and the core are homogeneous and that linear constitutive laws are valid. For the sake of generality, it was assumed that the core modulus of elasticity $E_{Cz}$ (in the *z*-direction, which is perpendicular to the facing plane) is independent of the core shear moduli $G_{Cxz}$ and $G_{Cyz}$ (moduli in the *x-z* and *y-z* planes, respectively). The case of elastic local buckling is considered (small deformations are assumed, and the effect of shortening the compressed element is ignored).

## 3. Determination of Critical Stress

The energy approach was employed to determine the critical load $p_{x}$ and the corresponding critical (wrinkling) stress. As in [2,12], core deformations in the longitudinal direction (along the x-direction) were not included in the energy equilibrium equation. The justification for this approach can be found, among other sources, in [23].

Assume that the facing deformation $w_{F}(x,y)$ has a sinusoidal form in the *x*-direction as follows:

(1) wFx,y=Wsin πxl·fy,

where W denotes the amplitude of deformation, and l is the length of the half-wave of the sine function in the *x*-direction. The facing band is very long (Figure 1 and Figure 2), and the length l is not initially known and will be determined during the assessment of the critical load $p_{x}$. The function $f\left(y\right)$, which represents the variation in deformation in the *y*-direction, is given here in an arbitrary form to obtain a potential general solution. The form of the function $f\left(y\right)$ should reflect the boundary conditions established for the longitudinal edges of the facing band. In the simplest form, corresponding to a simply supported band, $f\left(y\right)=\sin\frac{\pi y}{b}$.

The core deformation $w_{C}\left(x,y,z\right)$ disappears exponentially (k>0). This justifies the rapid disappearance of these deformations at the core depth:

(2) wCx,y,z=wFx,ye−kz=We−kzsin πxl·fy.

The following energy balance equation should be satisfied for the system shown in Figure 2b:

(3) $$U_{F}+U_{C}=U_{P}.$$

Here, the symbols denote the following:

$U_{F}$—the strain energy of the facing due to bending,

$U_{C}$—the strain energy of the core,

$U_{P}$—the work performed by applied load $p_{x}$.

Equation (3) can be written by taking into account the bandwidth b and the half-wavelength l of the sinusoid (increasing the integration length to 2l does not change the final solution). The strain energy of the facing, which should be treated as a plate, is expressed as follows:

(4) $$U_{F}=\frac{1}{2}B_{F}\int_{0}^{b}\int_{0}^{l}\left[{\left(\frac{\partial^{2}w_{F}}{\partial x^{2}}+\frac{\partial^{2}w_{F}}{\partial y^{2}}\right)}^{2}-2\left(1-\nu_{F}\right)\left(\frac{\partial^{2}w_{F}}{\partial x^{2}}\frac{\partial^{2}w_{F}}{\partial y^{2}}-{\left(\frac{\partial^{2}w_{F}}{\partial x\partial y}\right)}^{2}\right)\right]dxdy.$$

For the facing thickness $t_{F}$, the modulus of elasticity of the facing material $E_{F}$, and the Poisson ratio $\nu_{F}$, the bending stiffness (per unit width) is expressed as follows:

(5) $$B_{F}=\frac{E_{F}t_{F}^{3}}{12\left(1-\nu_{F}^{2}\right)}.$$

The work performed by the external load $p_{x}$ is expressed as follows:

(6) $$U_{P}=\frac{1}{2}\int_{0}^{b}\int_{0}^{l}p_{x}{\left(\frac{\partial w_{F}}{\partial x}\right)}^{2}dxdy.$$

The strain energy of the core consists of three terms corresponding to the *z*-direction deformation and two shear deformations is expressed as follows:

(7) $$U_{C}=\frac{1}{2}\int_{0}^{\infty }\int_{0}^{b}\int_{0}^{l}\left(\frac{1}{E_{Cz}}\sigma_{Cz}^{2}+\frac{1}{G_{Cxz}}\tau_{Cxz}^{2}+\frac{1}{G_{Cyz}}\tau_{Cyz}^{2}\right)dxdydz.$$

The normal stress $\sigma_{Cz}$ and the shear stresses $\tau_{Cxz}$ and $\tau_{Cyz}$ in the core can be expressed using partial derivatives of Equation (2) as follows:

(8) $$\sigma_{Cz}=E_{Cz}\frac{\partial w_{C}}{\partial z}=-E_{Cz}kWe^{-kz}\sin\frac{\pi x}{l}\cdot f\left(y\right),$$

(9) $$\tau_{Cxz}=G_{Cxz}\frac{\partial w_{C}}{\partial x}=G_{Cxz}\frac{\pi }{l}We^{-kz}\cos\frac{\pi x}{l}\cdot f\left(y\right),$$

(10) $$\tau_{Cyz}=G_{Cyz}\frac{\partial w_{C}}{\partial y}=G_{Cyz}We^{-kz}\sin\frac{\pi x}{l}\cdot \frac{df\left(y\right)}{dy}.$$

After substituting Equations (4)–(10) into Equation (3) and several transformations, an expression for the load $p_{x}$ is obtained:

(11) $$p_{x}=\frac{E_{Cz}}{2}k\frac{l^{2}}{\pi^{2}}+\frac{G_{Cxz}}{2}\frac{1}{k}+\frac{G_{Cyz}}{2}\frac{1}{k}\frac{l^{2}}{\pi^{2}}\frac{D}{C}+B_{F}\frac{l^{2}}{\pi^{2}}\left[{\left(\frac{\pi }{l}\right)}^{4}+\frac{E}{C}+2{\left(\frac{\pi }{l}\right)}^{2}\frac{D}{C}\right],$$

in which,

(12) $$C=\int_{0}^{b}{\left(f\left(y\right)\right)}^{2}dy,$$

(13) $$D=\int_{0}^{b}{\left(\frac{df\left(y\right)}{dy}\right)}^{2}dy,$$

(14) $$E=\int_{0}^{b}{\left(\frac{d^{2}f\left(y\right)}{{dy}^{2}}\right)}^{2}dy.$$

The condition for such a simplified solution is as follows:

(15) $${\left.\frac{df}{dy}\cdot f\left(y\right)\right|}_{0}^{b}=0.$$

To satisfy condition (15), it is sufficient for the function $f\left(y\right)$ or its first derivative to be zero at the edges of the facing band, which aligns with expected boundary behavior. Of note, the terms C, D, and E have different units: C [m], D [m^−1^], and E [m^−3^].

In Equation (11), aside from the material constants and the expressions that depend on the assumed function $f\left(y\right)$, there are only two unknowns, namely k and l. The critical load is obtained by simultaneously satisfying two equations representing the optimal conditions:

(16) $$\left\{\begin{array}{c} \frac{\partial p_{x}}{\partial l}=0\\ \frac{\partial p_{x}}{\partial k}=0. \end{array}\right.$$

If the parameters k and l are determined from Equation (16), the critical stress is obtained from (11), and the corresponding wrinkling stress is expressed as follows:

(17) $$\sigma_{Fx}=\frac{p_{x}}{t_{F}}.$$

The presented solution to the problem is easy to apply. The general form of $f\left(y\right)$ allows for the analysis of the influence of different stiffnesses of the support for the facing band on the value of the wrinkling stress. Thus, one can obtain, among other things, the upper limit of critical stress, which is of great importance when making decisions regarding changes in the sandwich panel production program.

The presented approach is limited by the assumptions presented in Section 2. In the case of continuous core inhomogeneities located near the facing, this approach can be further developed as presented for the 2D problems in [22] or [23]. In the case of local delamination, the presented approach is inefficient; in such situations, 3D numerical models with definitions of damage initiation and stiffness degradation can be used.

## 4. Results and Discussion

### 4.1. The Form of the Deformation Function

So far, the form of the function $f\left(y\right)$, which describes the variation in the deformation field along the width of the facing band, has been presented in a general form. Naturally, the form of this function affects the critical load value. This study seeks to identify a continuous function satisfying the appropriate boundary conditions at the edges of the band. Recall that condition (15) is assumed, although the entire derivation of (11) can be performed without this assumption. However, the solution will be slightly more complex without this assumption. The function that results in the lowest critical load value is determined from an engineering point of view, as this is when the buckling of the facing band will occur. The simplest approach to the issue of defining the function $f\left(y\right)$ is to rely on solutions of the form of buckling of a compressed rod. The classic solution for a supported rod of length b is expressed as follows:

(18) $$f\left(y\right)=sin\frac{\pi y}{b}.$$

The assumption of a sinusoid with a larger number of half-waves

(19) $$f\left(y\right)=sin\frac{n\pi y}{b}$$

is primarily of a theoretical nature because for the case n>1 the critical load is greater than for n=1 and is not realized in practice.

The second solution is to adopt the form of a function corresponding to the deformation of a compressed rod that is fixed on both sides. This is an extreme case, but it indicates an upper limit of the critical load value. The appropriate function in this case is expressed as follows:

(20) $$f\left(y\right)=1-cos\frac{2\pi y}{b}.$$

**Figure 3:**
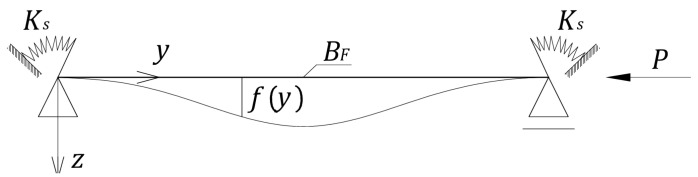
A rod with elastic rotational support at the ends.

It should be expected that in practice, the best representation of the facing band deformation will be the application of the deformation as for a simply supported rod, with an additional elastic restriction of rotation (Figure 3). In this case, the solution depends, among other things, on the relationship between the facing stiffness (5) and the stiffness of the elastic support per unit length of the band (hereinafter designated as $K_{s}$).

**Figure 4:**
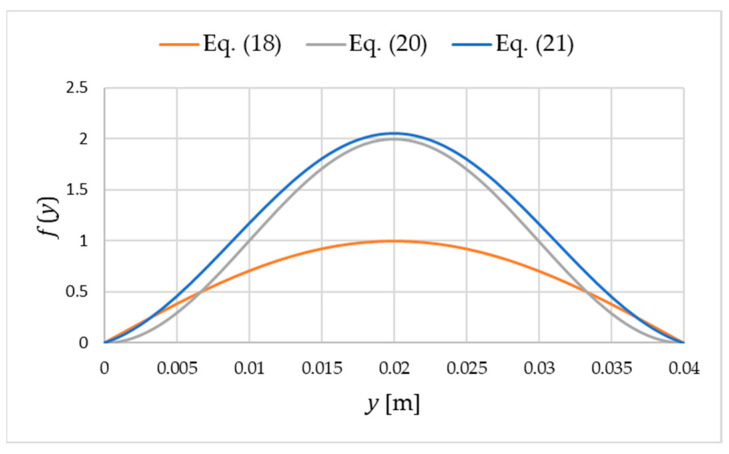
Examples of deformation functions (18), (20) and (21).

To determine the deformations shown in Figure 4, for given values of $B_{F}$, $K_{s}$, and b, one should determine $m={\left(P/B_{F}\right)}^{0.5}$ corresponding to buckling. Then, using any three independent boundary conditions, the following function is found:

(21) fy=1−cos mb+bm2BFKssinmb−mb·sinmy+cosmy−m1−cos mb+bm2BFKssinmb−mb+mBFKs·y−1.

Functions (18), (20) and (21) determined for b= 0.04 m are illustrated in Figure 4. To calculate Equation (21), $B_{F}=$ 0.0024038 kNm and $K_{s}=$ 1 kNm/m were also assumed. When analyzing a specific sandwich panel, the elastic support stiffness $K_{s}$ should be calibrated for the case under consideration (Section 4.3).

### 4.2. Wrinkling Stress as a Function of Width b

To maintain the generality of the considerations, the solution for Equation (19), i.e., the function that takes into account the possibility of multiple half-waves of the sine wave, is presented below. Equations (11) and (16) then take the following form:

(22) $$p_{x}=\frac{E_{Cz}}{2}\frac{kl^{2}}{\pi^{2}}+\frac{G_{Cxz}}{2}\frac{1}{k}+\frac{G_{Cyz}}{2}\frac{l^{2}}{k}\frac{n^{2}}{b^{2}}+B_{F}\pi^{2}l^{2}{\left(\frac{1}{l^{2}}+\frac{n^{2}}{b^{2}}\right)}^{2},$$

{(23a)k2−π2l2GCxz+GCyzn2l2b2ECz=0(23b)kECz+GCyzkn2π2b2+2BFπ4n4b4−1l4=0.

The optimal k and l are best determined numerically and then substituted into (22), after which the wrinkling stress is calculated from Equation (17). Since k>0 and half-wavelength l>0, the search procedure is as follows: from Equation (23a), k is determined as a function of l and substituted into Equation (23b). The search can be initiated from *l* = 0.001 mm with 1 mm steps, especially since a solution in the range of 35 mm is expected. For example, the bisection method can be used. The search was continued until an accuracy of l was established to 0.000001 mm. Such accuracy is not necessary because a change in l by 0.1 mm results in a change in the wrinkling stress of the order of 1 kPa.

**Figure 5:**
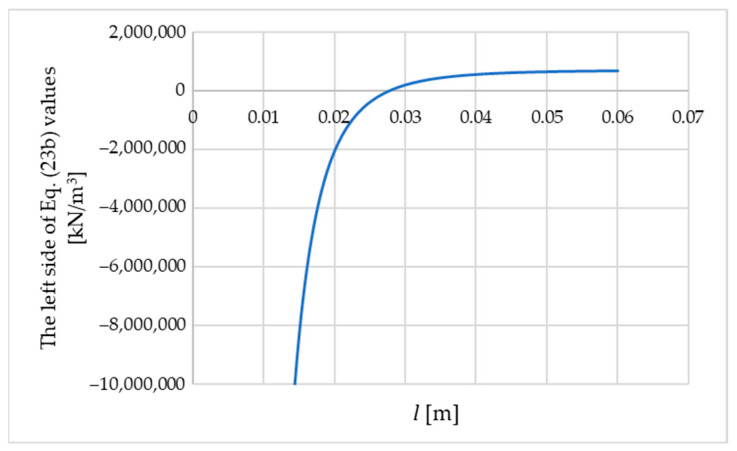
The values of the left side of Equation (23b) as a function of the variable l; b=0.04 m.

For n=1 and the following data, including $B_{F}=0.0024038$ kNm, $E_{Cz}=5.0$ MPa, and $G_{Cxz}=G_{Cyz}=2.38$ MPa, the optimal values of k and l are sought. There is no problem with local minima, as illustrated in Figure 5, which presents the values of the left-hand side of Equation (23b) as a function of the variable l (after substituting k from Equation (23a)).

**Figure 6:**
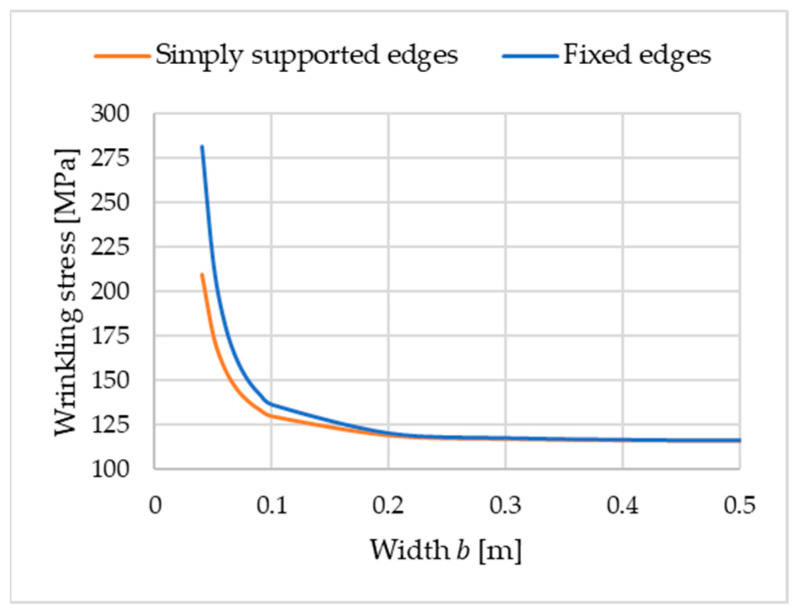
Wrinkling stress as a function of width b.

Figure 6 illustrates the critical stress as a function of the facing bandwidth b. The graph corresponding to Equation (19) is described as “simply supported edges”. Of note, the parameters assumed above correspond to typical values of manufactured sandwich panels.

Proceeding similarly as noted above, for the more general form of Equation (20) $f\left(y\right)=cos\frac{2n\pi y}{b}-1$, the load function is expressed as follows:

(24) $$p_{x}=\frac{E_{Cz}}{2}\frac{kl^{2}}{\pi^{2}}+\frac{G_{Cxz}}{2}\frac{1}{k}+\frac{G_{Cyz}}{2}\frac{l^{2}}{k}\frac{4n^{2}}{3b^{2}}+B_{F}\pi^{2}l^{2}\left(\frac{1}{l^{4}}+\frac{4n^{4}}{3b^{2}l^{2}}+\frac{16n^{4}}{3b^{4}}\right).$$

The optimality conditions are obtained as follows:

(25) $$\left\{\begin{array}{c} k^{2}-\frac{\pi^{2}}{l^{2}}\frac{G_{Cxz}+G_{Cyz}\frac{4n^{2}l^{2}}{3b^{2}}}{E_{Cz}}=0\\ kE_{Cz}+\frac{G_{Cyz}}{k}\frac{4n^{2}\pi^{2}}{3b^{2}}+2B_{F}\pi^{4}\left(\frac{16n^{4}}{{3b}^{4}}-\frac{1}{l^{4}}\right)=0. \end{array}\right.$$

The solution in the form of critical stress as a function of the facing bandwidth b is shown in Figure 6 and marked as “fixed edges”.

Several important conclusions can be drawn from the analysis of the results presented in Figure 6. First, changing the bandwidth b to a range of up to 0.1 m has no significant effect on critical stress. The load-bearing capacity of the band (and consequently also of the sandwich panel) increases only when the width of the band is less than 0.1 m, and this is essentially independent of the method used to support the band’s edges. The case of elastically supported longitudinal edges (corresponding to Equation (21) and Figure 3) falls between the two cases shown in Figure 6.

A comparison of the wrinkling stresses for the simply supported edges and fixed edges of the band shows that, as expected, the stresses are higher for the fixed edges. More importantly, for the given material parameters and specified static scheme, the wrinkling stress has a specific value. For b=0.04 m, with the simply supported edges of the band, the wrinkling stress is 209.38 MPa (l=0.02754 m, k=95.58 1/m). For fixed edges, it is 281.71 MPa (l=0.02292 m, k=113.43 1/m). Fixing the edges of the band increased the wrinkling stress by 82.33 MPa (39.3%) compared to the classical solution obtained from the literature (simply supported case).

In the case of elastic rotational restraint at the band edges, the wrinkling stress value will fall between the cases of simply supported and fixed edges. Therefore, sandwich panel manufacturers can obtain a higher wrinkling stress by slightly reducing the width of the band or modifying (improving) the material parameters. Of note, the stress values obtained in the presented examples are consistent with the order of stress values obtained in experimental tests. In the case of good-quality sandwich panels, the wrinkling stress of the analyzed facing band reaches a value of approximately 280 MPa. In experimental studies, this range of wrinkling stresses was obtained for deep-profiled panels with a thickness of 100–160 mm and a continuous polyisocyanurate foam core. Relevant information can be found in [24].

### 4.3. Band with Elastic Support of the Longitudinal Edges

When the optimality conditions in Equation (16) are applied to Equation (11), the following is obtained:

(26) $$\left\{\begin{array}{c} k^{2}-\frac{\pi^{2}}{l^{2}}\frac{G_{Cxz}+G_{Cyz}\frac{l^{2}}{\pi^{2}}\frac{D}{C}}{E_{Cz}}=0\\ kE_{Cz}+\frac{G_{Cyz}}{k}\frac{D}{C}+2B_{F}\left(\frac{E}{C}-\frac{\pi^{4}}{l^{4}}\right)=0. \end{array}\right.$$

**Figure 7:**
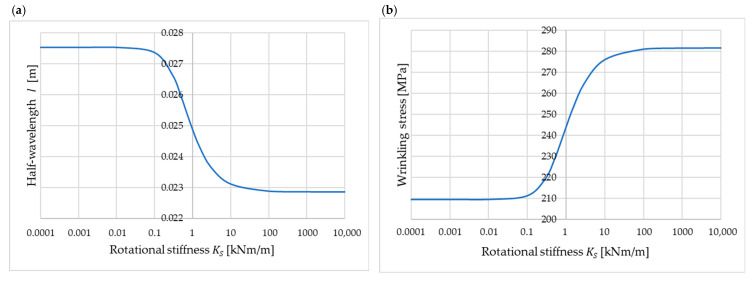
Influence of the rotational stiffness of the support $K_{s}$ on (**a**) half-wavelength l and (**b**) wrinkling stress.

If the parameter m is found, then using (21), C, D and E are determined from Equations (12)–(14). The rotational stiffness $K_{s}$ significantly influences the form of Equation (21), and therefore also the half-wavelength l and the wrinkling stress, as shown in Figure 7. Example calculations were performed for b= 0.04 m and the other parameters identical to those in Section 4.2.

The classical solution for simply supported longitudinal edges, known from the literature, corresponds to $K_{s}=0$. The results obtained for very low stiffness ($K_{s}<0.01$ kNm/m) are consistent with the (classical) case of simply supported edges. For high rotational stiffness ($K_{s}>100$ kNm/m), the results are consistent with the case of fixed longitudinal edges (see Figure 6 for b= 0.04 m). The half-wavelength l and the wrinkling stress are, however, sensitive to small changes in the stiffness $K_{s}$ in the range from 0.1 kNm/m to 10 kNm/m. The relationships presented in Figure 7 can be used to calibrate the model that takes into account the elastic support of the longitudinal edges of the considered band. It is also worth clearly emphasizing that, as illustrated in Figure 6, with the increase in width b, the influence of the stiffness of the longitudinal edge support on the wrinkling stress decreases.

### 4.4. Parametric Analysis

**Figure 8:**
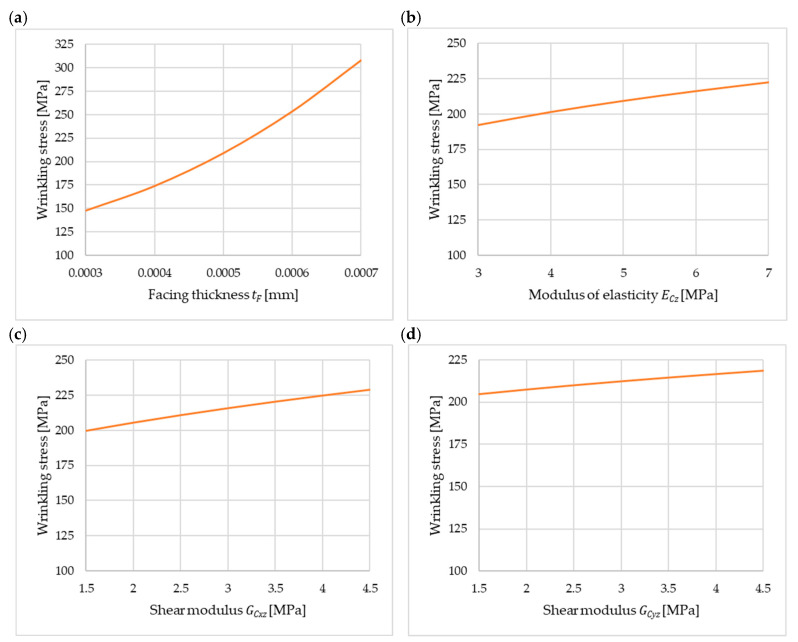
Stress wrinkling the facing band as a function of variation in (**a**) facing thickness $t_{F}$, (**b**) modulus of elasticity of the core $E_{Cz}$, (**c**) shear modulus of the core $G_{Cxz}$, and (**d**) shear modulus of the core $G_{Cyz}$.

To provide a more comprehensive analysis of the obtained solution, the corresponding parametric analysis is presented below. A case of simply supported longitudinal edges is considered (Equation (19)). Figure 8 presents the values of the wrinkling stress as a function of the variation in the facing thickness $t_{F}$ in the range from 0.30 mm to 0.70 mm (Figure 8a), the modulus of elasticity of the core $E_{Cz}$ in the range from 3.0 MPa to 7.0 MPa (Figure 8b), and the shear moduli $G_{Cxz}$ and $G_{Cyz}$ in the range from 1.5 MPa to 4.5 MPa (Figure 8c and Figure 8d, respectively). It is worth noting that in practice, moduli values do not assume arbitrary values. They are related to the material’s density, temperature, and even production process parameters. The calculations were performed for a bandwidth of b=0.04 m. For the presented parametric analyses, in the case of variation in one of the parameters, the remaining parameters were set as given in Section 4.2.

Most of the observations made from Figure 8 are consistent with expectations. The higher the core stiffness value is, the higher the wrinkling stresses. Wrinkling stresses are moderately sensitive to the change in the $E_{Cz}$, $G_{Cxz}$, and $G_{Cyz}$ modules. However, it is worth noting that the critical stresses clearly increase with the increase in the facing thickness (Figure 8a). As expected, the critical load must be greater because the thickness of the facing is greater; however, the significant increase in stress is somewhat surprising. This is particularly noteworthy because the standard [25] assumes an inverse relationship, i.e., when converting the wrinkling stress from a thinner to a thicker facing according to standard procedures, the wrinkling stress decreases.

## 5. Conclusions

The problem of local instability (wrinkling) of the facing band of a sandwich panel was analyzed in this study. The facing band rests on a shear-deformable core and can be considered supported at its edges due to the deep profiling of the facing. Both the support of the band on the core and the support of the band edges stabilize the band deformations. In this study, the case of uniaxial compression of the facing band, which is caused by typical mechanical actions, was considered.

In the first part of this paper, an energy approach was applied to the problem. This enabled the determination of the compressive load function of the facing band and the evaluation of optimal conditions for evaluating the wrinkling stress. The solution was presented in a general form for an arbitrary function describing the deformation of the facing band. The solution was obtained by assuming elastic local buckling. In cases of complex postcritical behavior or the occurrence of plastic flow effects, a more advanced model should be employed.

In the next stage of considerations, the derived equations were used, and exemplary solutions for the basic forms of the deformation function were presented. The presented theoretical approach enabled us to demonstrate that a significant increase in wrinkling stresses occurs for a bandwidth of less than 0.10 m. In addition, values corresponding to different support conditions of the band were obtained because the range of wrinkling stresses obtained in practice was known. To the authors’ knowledge, the results presented have not been previously obtained based on theoretical considerations.

Based on the derived equations, an analysis of the wrinkling stress variation as a function of the change in the facing thickness and parameters describing the core stiffness was performed. One observation is that wrinkling stress increases strongly with increasing thickness of the facing. This relationship, although logical, is inconsistent with the current normative provisions concerning sandwich panels.

The considerations presented in the article explain the issue of the influence of bandwidth on wrinkling stress. The derived equations can be directly used when planning a change in the production program of sandwich panels. The analyses presented indicate both the real possibilities of improving the load-bearing capacity of sandwich panels and the limitations related to increasing this capacity.

## Data Availability

The data presented in this study are available from the corresponding author upon request.

## References

[1] Hoff N.J., Mautner S.E. (1945). Buckling of Sandwich Type Panels. J. Aeronaut. Sci..

[2] Plantema F.J. (1966). Sandwich Construction: The Bending and Buckling of Sandwich Beams, Plates and Shells.

[3] Allen H.G. (1969). Analysis and Design of Structural Sandwich Panels.

[4] Birman V., Bert C.W. (2004). Wrinkling of composite-facing sandwich panels under biaxial loading. J. Sandw. Struct. Mater..

[5] Koissin V., Shipsha A., Skvortsov V. (2011). Wrinkling in sandwich panels—An analytical approach. J. Sandw. Struct. Mater..

[6] Fagerberg L., Zenkert D. (2005). Effects of anisotropy and multiaxial loading on the wrinkling of sandwich panels. J. Sandw. Struct. Mater..

[7] Birman V., Jeffrey L. (2020). Wrinkling in sandwich panels with bi-modular core. J. Appl. Math. Mech..

[8] Hohe J., Librescu L. (2008). Recent results on the effect of the transverse core compressibility on the static and dynamic response of sandwich structures. Compos. B Eng..

[9] Birman V. (2004). Dynamic wrinkling in sandwich beams. Compos. B Eng..

[10] Murčinková Z., Živčák J., Zajac J. (2020). Experimental study of parameters influencing the damping of particulate, fibre-reinforced, hybrid, and sandwich composites. Int. J. Mater. Res..

[11] Phan C.N., Bailey N.W., Kardomateas G.A., Battley M.A. (2012). Wrinkling of sandwich wide panels/beams based on the extended high-order sandwich panel theory: Formulation, comparison with elasticity and experiments. Arch. Appl. Mech..

[12] Birman V., Vo N. (2017). Wrinkling in sandwich structures with a functionally graded core. J. Appl. Mech..

[13] Su W., Liu S. (2025). New method for predicting the wrinkling stress in sandwich panels. Arch. Appl. Mech..

[14] Ginot M., Bouvet C., Castanié B., Serra J., Mahuet N. (2023). Local buckling on large sandwich panels used in light aviation: Experimental setup and failure scenarios. Compos. Struct..

[15] Ren X., Zhang S., Wu Z. (2023). A strategy resisting wrinkling of sandwich structures reinforced using functionally-graded carbon nanotubes. Chin. J. Aeronaut..

[16] Gdoutos E.E., Daniel I.M., Wang K.A. (2003). Compression facing wrinkling of composite sandwich structures. Mech. Mater..

[17] Steineck S., Lange J. (2024). Material Behavior of PIR Rigid Foam in Sandwich Panels: Studies beyond Construction Industry Standard. Materials.

[18] Pradhan E.M., Lange J. (2024). Warping Torsion in Sandwich Panels: Analyzing the Structural Behavior through Experimental and Numerical Studies. Materials.

[19] Indreș A.I., Constantinescu D.M., Mocian O.A. (2021). Bending behaviour of 3D printed sandwich beams with different core topologies. Mater. Des. Process. Commun..

[20] Atalay Kalsen T.S., Karadağ H.B., Eker Y.R. (2023). The Out-Of-Plane Compression Behavior of In Situ Ethylene Vinyl Acetate (EVA)-Foam-Filled Aluminum Honeycomb Sandwich Structures. Materials.

[21] Hassinen P., Misiek T. (2012). Einfluss von Inhomogenitäten im Kernwerkstoff von Sandwichelementen auf die Tragfähigkeit. Stahlbau.

[22] Pozorski Z., Pozorska J. (2022). Influence of the Heterogeneity of the Core Material on the Local Instability of a Sandwich Panel. Materials.

[23] Pozorski Z., Pozorska J., Kreja I., Smakosz Ł. (2021). On Wrinkling in Sandwich Panels with an Orthotropic Core. Materials.

[24] Pozorski Z. (2016). Sandwich Panels in Civil Engineering, Theory, Testing and Design.

[25] (2013). Self-Supporting Double Skin Metal Faced Insulating Panels—Factory Made Products—Specifications.

